# Skin microbiome influences the progression of cutaneous squamous cell carcinoma through the immune system

**DOI:** 10.1186/s12957-025-03791-5

**Published:** 2025-04-09

**Authors:** Zijian Zhang, Lili Liang, Xiaoke Jiang, Jixuan Shan, Siying Li, Jie Liu, Qinyi Dong, Xinman Wang, Han Zhang

**Affiliations:** 1 Shanxi University of Chinese Medicine, Taiyuan, China; 2 Department of Dermatology, Shanxi Provincial People’s Hospital (Fifth Hospital) of Shanxi Medical University, Taiyuan, China; 3 Department of Dermatology, Fenyang Hospital of Shanxi Province, Fenyang, China

**Keywords:** Cutaneous squamous cell carcinoma, Skin, Microbiome, Immune system, Skin cancer

## Abstract

Cutaneous squamous cell carcinoma (cSCC) is a type of skin tumor that develops in the epithelial cells. This disease has the second highest incidence of human skin cancers, with a high metastatic rate. While ultraviolet radiation significantly contributes to the genomic changes that support cSCC development, the dysbiosis of the skin microbiome and influence of the immune system also play important roles in this process. In this review, we discuss the effects of skin microbes and their metabolites on the immune system, including innate immune cells, T cells, and cytokines. We also discuss how *Staphylococcus aureus* and human papillomavirus can affect cSCC by impacting the immune system. Furthermore, we explore the antagonism of symbiotic microorganisms with cSCC-associated pathogens and their potential as novel therapeutic modalities.

## Background

Cutaneous squamous cell carcinoma (cSCC) accounts for approximately 20% of skin tumor cases, second only to basal cell carcinoma [1]. The incidence of SCC increased by approximately 310% in 195 countries worldwide between 1990 and 2017 [2]. At present, the main treatment methods for cSCC are surgery, radiotherapy, and photodynamic therapy, with some immunosuppressants and targeted drugs also being used. However, no safe and effective treatment is available for patients who cannot undergo surgery and those who are immunosuppressed.

**Fig. 1:**
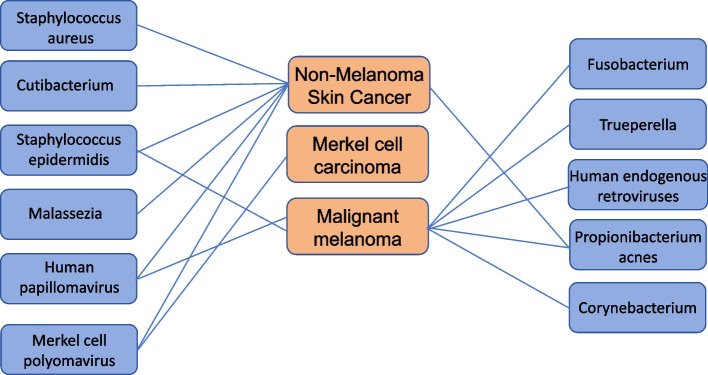
Multiple skin microbiotas have been associated with skin cancers (including non-melanoma, malignant melanoma, and Merkel cell carcinoma). Compared with healthy skin, there were differences in the presence of *Human papillomavirus*, *Staphylococcus aureus*, *Staphylococcus epidermis*, Malazzia, *Cutibacterium*, *Propionibacterium acnes*, and *Merkel cell polyomavirus* in non-melanoma skin. Merkel cell polyomavirus is also the main cause of Merkel cell carcinoma. *Human papillomavirus*, *Fusobacterium*, *Trueperella*, *Human endogenous retroviruses*, *Propionibacterium acnes*, and *Corynebacterium* in malignant melanoma were significantly different from those in normal skin

Human skin is a habitat for a variety of microorganisms that are present throughout the surface of skin attachments. They maintain normal skin function through various actions, including preventing pathogen invasion and interacting with the immune system [3]. Actinic keratosis (AK) is widely recognized as a precancerous lesion of cSCC. Studies have demonstrated clear differences in the microbial composition and abundance in AK, cSCC, and healthy skin, although a causal relationship between bacterial colonization and AK and cSCC progression has not been established. However, the increased abundance of *Staphylococcus aureus* (*S. aureus*), an important pathogenic bacteria in cSCC, was directly proportional to the disease progression. Microbiota can regulate many aspects of the immune system. For example, skin microbiota can control the expression patterns of antimicrobial peptides (AMPs), β-defensin, and other innate immune factors, thus affecting the course of skin diseases [4] (Fig. 1).

Some skin symbiotic microorganisms can trigger the host immune system to regulate pathogen colonization. *Staphylococcus epidermidis* (*S. epidermidis*) limits invasion of the fungal pathogen Candida albicans by inducing the production of interleukin (IL)− 17A and CD8^+^ T cells. These findings suggest that skin symbiotic microbes have therapeutic potential [5].

Overall, skin microbes play an important role in cSCC pathogenesis and progression. Here, we review the interactions between cSCC-related skin microbes and skin immunity, then discuss the prospect of applying skin symbiotic microorganisms for treating cSCC in the future.

## cSCC

cSCC is one of the most common malignant tumor types affecting the skin. Histopathological examination is the gold standard for diagnosing cSCC, which manifests as an epidermal erosion or ulcer, irregular tumor cells with infiltrating growth in the dermis, and atypical squamous cells with irregular nuclei, deep staining, and loss of intercellular bridge. Angular beads and abnormal fission phases are also seen [6].

cSCC is a multi-factorial disease. Epidermal cell genomic alterations from ultraviolet (UV) radiation play an important role in the early onset of cSCC [7]. Furthermore, fair skin, chemical carcinogens, human papillomavirus (HPV) infection, and immunosuppression are also risk factors for cSCC onset and progression. Multiple gene networks, such as tumor suppressor p53 (TP53), CDKN2 A, and NOTCH1, as well as molecular pathways, such as PI3 K/AKT/mTOR, are related to cSCC pathogenesis [8]. TP53 gene mutations are the most common and frequent genomic alterations observed in cSCC, with a large number of UV-induced TP53 point mutations detected in early sequencing studies of cSCC [9]. In addition, mutations in the genes encoding p53 and NOTCH pathway-associated proteins were prevalent in cSCC, with approximately 75% of cSCC cases having UV-signature loss of function mutations in NOTCH1 and NOTCH2 [10, 11]. This suggests that NOTCH may be an early mediator involved in the transition of normal keratinocytes to a precancerous state. CDKN2 A was significantly associated with tumor invasiveness. CDKN2 A locus mutations were detected in 24% to 45% of sporadic cSCC cases, suggesting that CDKN2 A plays an important role not only in precancerous lesion formation, but also in the progression from precancerous lesion to aggressive cSCC [12].

Surgery is the first-line treatment for cSCC, where the tumor can be surgically removed to prevent metastasis, and in most cases, the prognosis for cSCC is good. However, approximately 5% to 10% of cSCC cases progress to locally advanced or metastatic disease [13], and due to the usually large scope of surgical resection, some patients do not undergo surgical resection, and these patients need to be evaluated by a multidisciplinary team before alternative treatment options are selected. Radiation therapy and chemotherapy are also used as important methods of adjuvant therapy for treating cSCC, which can help control local lesions, prevent cancer spread, and reduce tumors size. Although these approaches are suitable for patients who are not candidates for surgical resection, radiotherapy and chemotherapy have significant side effects [13].

In recent years, targeted therapy and immunotherapy have been increasingly applied for cSCC. The epidermal growth factor receptor (EGFR) inhibitor cetuximab is an example of a systemic therapy that can be used to treat cSCC [14]. However, the efficacy of EGFR inhibitors has been slightly lower than expected, with some targeted therapies often disrupting skin homeostasis and causing side effects. A number of inhibitors targeting programmed cell death protein 1 (PD- 1) have been used to treat cSCC. For example, cemiplimab has been shown to be effective in patients with advanced cSCC and metastatic disease with normal immune function [15]. Despite these observations, immunotherapy has higher treatment conditions and is not suitable for patients with immunosuppression, such as solid organ transplantation (SOT) patients. Therefore, more adaptable treatment options are needed, with new inhibitors that are less invasive and have fewer associated side effects.

Recent evidence based on observational and microbiome sequencing studies suggests that dysregulation of the skin microbiome is related to cSCC pathogenesis [16–20]. Many microorganisms, such as viruses, fungi, and bacteria, colonize the surface of human skin and glands. The composition and distribution of different microorganisms maintain a dynamic balance within a certain range, regulating the skin environment of the host through proliferation, secretion, and other mechanisms. During the carcinogenesis process, changes in skin tissue morphology and metabolism also cause alterations to the microbial habitat. This alteration in skin microbiota composition favors the colonization of microorganisms that may contribute to carcinogenesis, thus leading to the continuous carcinogenic transformation of the skin [16].

The immune system plays a key role in cSCC pathogenesis and progression. Clinical observational studies have found that the risk of cSCC in patients with organ transplantation and long-term immunosuppressive treatment is 100 times higher than that of the general population. In addition, immunosuppression may also cause cSCC to be more aggressive [21].

With the development of microbial detection technology and increasing research on the immune system, many studies have started focusing on the interactions between them. Different types of skin microorganisms have varied regulatory effects on the host immune system during skin cancer development and progression. Therefore, investigating the interactions between skin microbes and the immune system and their involvement in cSCC pathogenesis and progression is worthy of further exploration.

## The relationship between the skin microbiota and immune system

An increasing number of researchers are examining the impact of the skin microbiota on the immune system. Host innate immune cells can sense skin microbes and take up their metabolites, while host systemic immune factors can significantly influence the composition of microbes on the skin. For example, immunodeficient patients have significantly different skin microbial compositions compared with normal individuals [18].

Microorganisms colonizing neonatal skin contribute to bacterial antigen presentation by inducing symbiosis-specific regulatory T cells to interact with CD301 type II conventional dendritic cells (DCs) to improve host immune tolerance. Innate-like T (iNKT) cells, including natural killer T (NKT) cells and mucosal-associated invariant T (MAIT) cells, are involved in the interaction between the microbiome and immune system. Early-life exposure to nonpeptide metabolites of the skin microbiome presented by major histocompatibility complex (MHC) class Ib molecules plays a decisive role in the development of these two T cell classes [22]. In addition, the skin microbiome maintains the skin’s barrier function by regulating immune cells and keratinocytes. Toll-like receptor (TLR) 2 and stem cell factors on keratinocytes, as well as lipoteichoic acid from gram-positive bacteria, can work together to promote mast cell recruitment in the skin [23]. For example, *S. epidermidis* can inhibit TLR3-triggered skin inflammation in a TLR2-dependent manner via lipoteichoic acid produced by keratin-forming cells [24]. Skin bacteria can also induce host cells to secrete factors, such as conductin, β-defensin, lipids, and hyaluronic acid, to enhance antimicrobial immunity and support histone deacetylase-mediated reduction of tissue-specific inflammation. Some human skin commensals can also promote the barrier function and accelerate skin repair by activating the keratinocyte aryl hydrocarbon receptor (AhR) [25]. Thus, these positive regulatory effects of skin microorganisms on the immune system has therapeutic implications in cSCC.

Furthermore, adaptive immunity is an important part of the human immune system. Unlike infection-induced responses, T and B cell responses to the microbiome and their accumulation in tissues do not occur only in the context of inflammation. In skin with a normal barrier function, colonization by *S. epidermidis* activates and aggregates antigen-specific T cells to secrete IL- 17 and interferon (IFN)-γ in an IL- 1R and MYD88-dependent manner under homeostasis [26], as well as promotes AMP production in an IL- 17 A-dependent manner. This leads to increased protection against subsequent infections. In response to metabolites produced by microbes, microbially induced cytokine responses, MAIT cells, and γδ T cells are also enriched within the skin and contribute to wound repair [27]. Specific bacterial branches can also promote the induction and tissue accumulation of H2-M3-restricted CD8^+^ T cells, which support tissue repair by releasing IL- 13 [28, 29]. They also promote keratinocyte production of AMPs, thus increasing heterologous protection against subsequent fungal infections [30].

Conversely, in mice with skin barrier breakdown, *S. aureus* colonization can increase the expression levels of the pro-inflammatory cytokines IL- 1β, IL- 6, and tumor necrosis factor α (TNF-α) [31]. Additionally, delta-toxin secreted by *S. aureus* can induce dermal mast cell debudding and promote the helper T lymphocyte (Th) 2 response [32]. SAg, a superantigen expressed by *S. aureus*, significantly activates T cells, interacts with mast cells, induces T cells to secrete IL- 31 [33], inhibits keratinocyte differentiation, and downregulates filament polyprotein expression. In addition, phenol-soluble regulator (PSM) α produced by *S. aureus* can trigger IL- 36R/MyD88 signaling, with epidermal keratinocyte-derived IL- 36 stimulating T cells and leading to excessive production of IL- 17 [34]. IL- 17 is a key inflammatory mediator in a variety of autoimmune, allergic, and infectious diseases, with numerous studies also demonstrating the tumorigenic role of high IL- 17 levels in human cancers [35–38].

In addition, skin sites in immunocompromised individuals are more susceptible to colonization by pathogenic bacteria than in those with normal immune function. They also display decreased microbial diversity [18]. For example, certain patients have primary immune deficiency, such as those with CXCR4-deficient WHIM syndrome [39], JAK3 deficiency [40], and GATA2 deficiency [41]. Because of reduced HPV-targeted cytotoxicity, NK cell count, CD8^+^ T cell count, CD4^+^ T cell lymphocytopenia, and other reasons [42], these patients are at risk of persistent HPV infection and high recurrence, with a greater probability of developing malignant disease [41]. Patients with CARD9 loss of function mutations show a selective susceptibility to a variety of superficial and invasive fungal diseases, including cutaneous mucocutaneous candidiasis, dermatophytosis, subcutaneous acidophilosis, deep dermatophytosis, and central nervous system candidiasis, and may progress to fatal lymph node and central nervous system infections [43]. Alves de Medeiros et al. [44] reported a family of homozygous R70 W CARD9 mutations with drug-resistant chronic skin and deep skin mycosis, as well as cutaneous mucosal and invasive candidiasis.

Moreover, SOT patients often receive immunosuppressive therapy to improve long-term graft and survival. However, this reduction in immune function results in approximately 70% of serious skin infections occurring in the first three months after transplantation, with fungal and viral infections being more frequent than bacterial infections [45]. Fungal infections are the most common skin infections within two years after SOT, with superficial mycosis occurring in about 60% of SOT. These are mainly caused by Candida albicans, Malazzia furs, cutaneous fungi, and Fusarium [46]. Fungal infections in immunosuppressed transplant recipients are often clinically more aggressive, malleable, and refractory to treatment than in the general population. Viral infections are the second most common. Data suggest that the occurrence of HPV-associated warts is related to the duration of immunosuppression. Specifically, 50% to 90% of patients will have warts 4 to 5 years after transplantation [47], with the reduced immune surveillance caused by immunosuppression leading to HPV reactivation and replication. Bacterial infections also occur in these individuals. Folliculitis, inflammation of the hair follicles mainly caused by *S. aureus*, is the most common bacterial infection in the early post-transplant period [48]. Impetigo is another bacterial disease that is common in SOT patients within the first year after transplantation [45]. This is a superficial bacterial infection caused primarily by *S. aureus* or hemolytic streptococcus. Erysipelas is an acute bacterial infection of the dermis and superficial lymphatic vessels, the common pathogen being Group A Streptococcus. Immunosuppression is another contributing factor to these infections in SOT patients [49]. In addition, human immunodeficiency virus (HIV) infection may also increase patient susceptibility to HPV. Additionally, interactions between the two viruses can result in decreased levels of key innate molecules, including B-defencin- 2 and thrombospondin, leading to an increased risk of HPV-induced malignancy [50]. In one report, adult HIV patients generally displayed higher rates of *S. aureus* colonization than the general population, up to 81% over the 1-year study period. They also had more frequent invasive *S. aureus* infections and bacteremia development than the HIV-negative controls [51].

## The skin microbiota interacts with the immune system and can influence the course of cSCC

The skin microbiota helps maintain the skin barrier function, regulating the immune system and defending against pathogens [52]. With this role, it can inhibit pathogen invasion through both AMP secretion and interactions with keratin-forming cells and immune cells [53]. Together, UV irradiation, immune system alterations, microbial system disorders, and chronic trauma can collectively promote cSCC development.

**Fig. 2:**
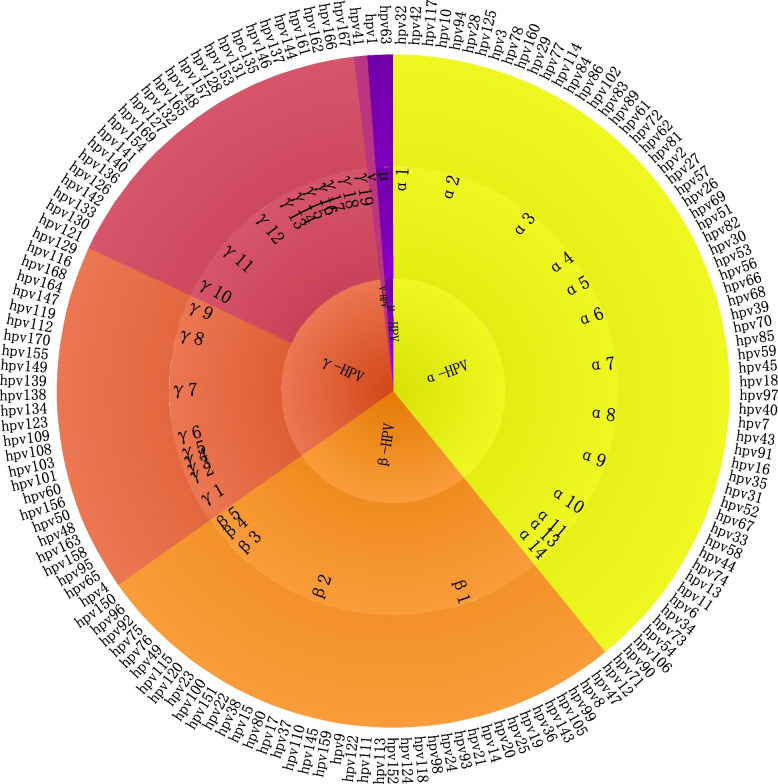
Major known HPV genera. HPV infection is an important factor in the pathogenesis of many squamous cell carcinomas, and more than 150 HPV genotypes have been fully sequenced, and HPV types are classified into five genera based on the nucleotide sequence of the L1 gene: α, β, γ, ν, and μ

HPV is a DNA virus that parasitizes humans, with HPV infections significantly contributing to the development of a variety of forms of SCC [54]. HPV can pass through small lesions in the skin and reach the basal layer of the epidermis, where its E4 protein can mediate and regulate viral particle release to infect cells in the basal layer. These infected cells can subsequently migrate from the basal layer upward to the superficial layer of the skin [55], which in turn leads to disease aggravation (Fig. 2).

HPV is categorized into five genera using the L1 nucleotide sequences (alpha, beta, gamma, mu, and nu). Both α-HPV and β-HPV are closely related to cSCC, with nearly half of patients presenting as high-risk α-HPV positive. Moreover, a large proportion of cSCC patients also have a history of other α-HPV-related diseases [56]. Among individuals with a typical immune system who tested positive for β-HPV, a 42% increased risk of developing cSCC was found. Serum antibody testing also supported this observation, with serological testing of 2,366 patients showing a positive association between SCC and β-HPV type [57]. In immunosuppressed organ transplant recipients, the ability to fight β-HPV is significantly reduced compared with β-HPV-specific T cells. Additionally, when the immune function of β-HPV-specific T cells is impaired, β-HPV and virus-positive malignant keratinocyte clearance is reduced, which further exacerbates the deterioration of cSCC [58].

Two HPV oncoproteins, E6 and E7, play important roles in cancer development and HPV-induced carcinogenesis. During this process, HPV E6 interacts with p53, disrupting the binding of p53 to its site-specific DNA sequences [59]. E6 can also inhibit the trans-activation of p53 response genes by interacting with CREB-binding protein (CBP)/p300 and hADA3, as well as by disrupting the stability of Tip60, thus maintaining continuous cell proliferation [60]. In addition, E6 can inhibit differentiation-induced apoptosis in human foreskin keratinocytes by regulating the expression patterns of the anti-apoptotic protein Bcl- 2 and pro-apoptotic protein Bax [61]. The E6 protein can also lead to cancer development by regulating a variety of signaling pathways. HPV- 16 E6 expression increases mTOR1 activity by upstream activation of mTOR2 and 3-phosphoinositol-dependent kinase 1 (PDK1), leading to AKT activation [62]. The PI3 K/AKT pathway is associated with cancer survival, with studies showing that HPV E6 can inactivate PTEN, leading to increased pAKT levels and enhanced cell proliferation. E6 targets Mastermind-Like (MAML) through the LXXLL motif and binds to MAML- 1 to inhibit Notch signaling, thereby delaying keratinocyte differentiation, while the combined inactivation of p53 and Notch promotes cell proliferation with high cell density [63]. This can thereby increase the possibility of infected cells developing into malignant tumors. The E6 protein does not act alone in the carcinogenic process, typically interacting with the E7 protein to promote cancer. E7 can inhibit the tumor suppressor gene retinoblastoma protein (pRb) 8 to promote cell proliferation [64]. E6 interferes with cell survival pathways and E7 promotes cell proliferation, with the combination of the two oncoproteins leading to an increased number of centrosomes in models of head and neck SCC and large, highly aggressive tumors [65].

Activin A, a member of the transforming growth factor-β (TGF-β) superfamily, is a growth and differentiation factor that promotes wound healing and skin morphogenesis [66]. Activin expression levels are upregulated in mouse and human skin wounds, as well as in cSCC. Its overexpression reduces the number of tumor suppressive γδ T cells and increases the number of protumorigenic regulatory T cells in the ear skin of HPV8 transgenic mice, which promotes the occurrence of skin tumors. In addition, there was an apparent increased presence of epidermal CD4^+^ and CD8^+^ T cells in the tumor-bearing skin of the mice in that study. However, the genetic depletion of CD4^+^ T cells did not reduce the protumorigenic effects of activin [67], potentially indicating that HPV and T cell perturbation alone are not sufficient to support cSCC development in the presence of activin overexpression. Strickley et al. [68]. reported that CD8^+^ T cell depletion in MmuPV1-colonized mice increased tumorigenicity following chemical carcinogenesis, suggesting that CD8^+^ T cells can mediate antitumor immunity induced by skin colonization with MmuPV1. CD8^+^ T cell depletion increased the MmuPV- 1 DNA levels in the skin of the mice and resulted in higher levels of antibodies targeting the E6, E7, MmuPV1, and L1 antigens, demonstrating that loss of T cell immunity against β-HPV can increase the viral load in the skin of immunosuppressed patients and lead to a higher risk of cSCC [69]. Dorfer et al. [70] used cyclosporin A (CsA) to inhibit calmodulin phosphatase and T cell activation. Compared with immunocompetent mice, the dorsal skin of mice that were immunosuppressed using CsA and infected with MmuPV1 displayed cSCC development.

**Fig. 3:**
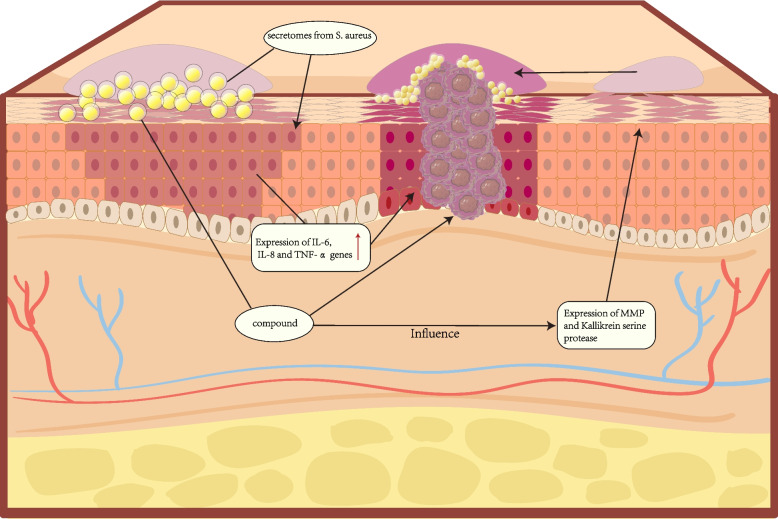
*Staphylococcus aureus* affects and drives the progression of cSCC of the skin. Staphylococcus aureus affects and drives the progression of cSCC of the skin in a variety of ways. Staphylococcus aureus secretion group can enhance the expression of IL- 6, IL- 8 and TNF-α genes in human keratinocytes, and promote the formation of tumor microenvironment in the skin. *Staphylococcus aureus* strains can secrete compounds similar to ultraviolet irradiation, which can promote the development of cSCC by expressing biomarkers of cSCC, while affecting the expression of MMP and kallikrein serine protease, reducing skin barrier function, damaging DNA repair and skin barrier function, and thus promoting epidermal hyperplasia and cSCC development

*S. aureus* is a common human-veterinary pathogen that can induce inflammatory reactions and a variety of diseases in humans and animals, making it a serious threat to human and animal life [71]. One study investigated the microbial communities associated with AK and SCC in longitudinal and cross-sectional sections. Their work revealed that the abundance of *S. aureus* differed significantly between AK and SCC patients and the experimental controls, with *S. aureus* playing a specific role in the progression of AK to SCC [16] (Fig. 3). Inflammation is an important mechanistic contributor to carcinogenesis. It has been suggested that *S. aureus* can lead to tumor formation by producing chronic inflammation, inducing various cytokines, including TNF-α, and activating NF-κB signaling [72]. Krueger et al. [73] reported that the secretomes of *S. aureus* strains isolated from photodamaged skin, AK, and SCC samples led to increased expression of IL- 6, IL- 8, and TNF-αin human keratinocytes. Moreover, IL- 8 and TNF-α gene expression levels were enhanced [74]. Interestingly, IL- 6, IL- 8, and TNF-α have procarcinogenic functions and are frequently overexpressed in many malignant tumor types [31]. Thus, the colonization of certain *S. aureus* strains may promote the development of a tumor-supportive microenvironment in the skin more than other strains. In addition, IL- 6 protein levels were significantly elevated after keratinized cells were treated with the SCC-associated *S. aureus* secretome, which also triggered a pronounced innate immune response in mouse skin in vitro. Thus, IL- 6-induced *S. aureus* skin colonization may result in a sustained accumulation of immune cells, which is associated with cancer progression [75].

Some *S. aureus* strains can secrete compounds similar to UV radiation, which can promote SCC progression through the expression of SCC biomarkers. They can also impair the DNA repair and skin barrier functions, leading to further oxidative stress and DNA damage in the skin. The *S. aureus* secretome isolated from AK and cSCC samples can affect the expression patterns of matrix metalloproteinases (MMPs) and the kinase-releasing enzyme serine protease, leading to extracellular matrix breakdown, reduced barrier function, and compromised skin barrier integrity. This thereby can promote epidermal hyperplasia and SCC development [74]. Human β-defensin- 2 (hBD- 2) is potentially involved in tumor formation by influencing tumor cell growth and migration, as well as by being induced by specific microbial responses. *S. aureus* was significantly associated with increased hBD- 2 expression levels in SCC samples, suggesting that *S. aureus* can induce specific expression of hBD- 2 to promote tumor cell growth and influence tumor progression [76].

## Potential treatment of cSCC by regulating the skin microbiome

**Fig. 4:**
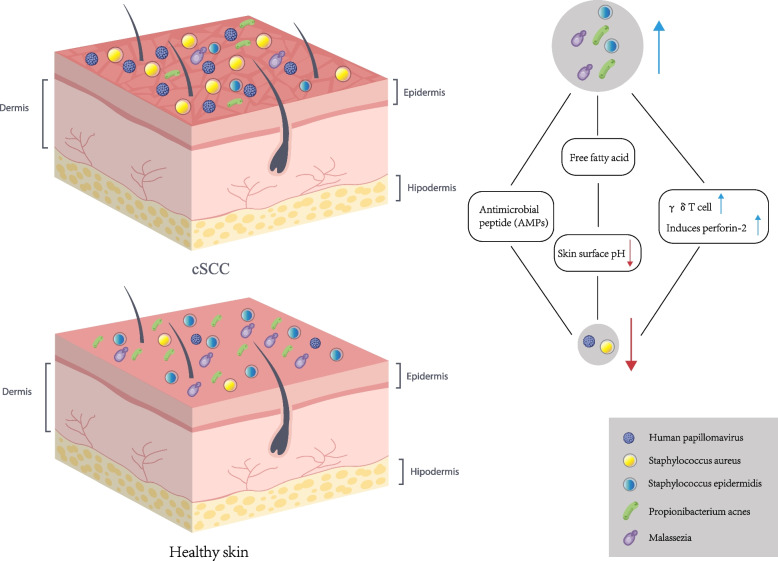
Regulation of skin microecology by skin symbiotic bacteria. Skin symbiosis has been studied, they have an impact on the immune microecology, and play a corresponding role in skin cancer is undoubtedly, they can produce free fatty acids, reduce the surface pH, form an acidic environment, inhibit the colonization of pathogenic bacteria. Some skin symbiotic bacteria have antagonistic properties with cSCC-associated pathogens, and they can secrete AMPs or antibacterial compounds to fight skin pathogens. In addition, it can activate γδT cells and up-regulate the expression of perforin- 2 in human skin, enhancing the ability to inhibit pathogens

Cutaneous symbiosis has been studied in recent years. Although the underlying mechanism is poorly understood, these bacteria clearly impact the immune microecology and play a corresponding role in skin cancer, which can affect the immune state through the production of metabolites. In addition, some skin symbiotic bacteria have antagonistic properties with cSCC-associated pathogens and can secrete AMPs or antimicrobial compounds to fight skin pathogens. (Fig. 4).

*Propionibacterium acnes* (*P. acnes*) is the most common commensal bacteria on human skin. *P. acnes* induces increased levels of essential lipids, such as triglycerides, ceramides, cholesterol, and free fatty acids, the synthesis of which plays an extremely important role in epithelial barrier formation and external microorganism homeostasis [77]. *P. acnes* also metabolizes the free fatty acids in sebum, lowering the surface pH and creating an acidic environment, which can help inhibit the colonization of pathogenic bacteria such as *S. aureus*.

As the skin progresses from a healthy state to AK to SCC, *P. acnes* levels decrease while *S. aureus* levels increase. Voigt et al. [19] studied the ratio of *P. acnes* to *S. aureus* and proposed that the two bacteria have antagonistic effects. Because the skin microecology is altered by tumors or malignant tissues, sebaceous gland loss is not conducive to *P. acnes* growth. This leads to the proliferation of pathogenic bacteria, such as *S. aureus*, which accelerates the AK transition into cSCC.

*S. epidermidis* is a gram-positive bacillus belonging to the normal flora. Unlike *S. aureus*, *S. epidermidis* is mostly non-pathogenic and usually maintains a benign relationship with the host, with a few strains causing septic infections in humans and animals [78]. *S. epidermidis* can play a protective and anti-tumor role by activating the immune system to fight cancer cells and compete with other potentially harmful bacteria. Nakatsuji et al. [79] reported that coagulase-negative staphylococcus displayed different degrees of antibacterial activity against *S. aureus* in patients with atopic dermatitis and healthy patients. They observed that *S. epidermidis* strains could produce new lantibiotics that inhibited *S. aureus* growth. In addition, *S. epidermidis* can produce strong selective AMPs against *S. aureus*. *S. epidermidis* can also activate γδT cells, upregulate perforin- 2 expression levels in human skin, and enhance the inhibition ability against *S. aureus* [80]. A recent study by Nakatsuji [81] showed that the *S. epidermidis* strain MO34 could reduce the incidence of UV-induced tumors in mice by producing 6-n-hydroxy-aminopurine (6-HAP). 6-HAP can induce DNA damage and apoptosis in human cancer cells and destroy chromosomes in epidermoid carcinoma [82]. Although the therapeutic effects of *S. epidermidis* in cSCC have not been demonstrated in clinical trials, another *S. aureus* strain with the same lantibiotics as *S. epidermidis*, Staphylococcus hominis A9 (ShA9), has been shown to reduce *S. aureus* levels and inhibit the expression of *S. aureus* toxins in phase I clinical trials. This resulted in improved skin lesions in patients with atopic dermatitis [83]. These findings suggest that *S. epidermidis* may be useful as a probiotic for treating cSCC.

*Malassezia* is the most abundant fungal community on mammalian skin, with 14 known species. All of these can infect or colonize the human body [84], forming the major eukaryotic microbial community on human skin. *Malassezia* is also associated with several common skin conditions, including florid chafing, seborrheic dermatitis, and other inflammatory skin diseases. Some studies have confirmed that they can exacerbate seborrheic dermatitis, dandruff, folliculitis, nail fungus, and psoriasis [85]. The genus *Malassezia* possesses a range of secreted hydrolases that are important mediators of microbial-host and intermicrobial interactions, participating in protein and lipid metabolism and altering the external skin environment [57]. The fungal communities detected in cSCC are dominated by *Serratia marcescens* and Bacillus restrictans [86]. Wood et al. [16] reported that the operational taxonomic units (OTUs) of *Serratia marcescens* were more commonly found in nonlesional skin than in precancerous AK of SCC. In the photodamaged nondiseased control samples collected from patients, the *Malassezia* OTU abundance was greater than that in AK and SCC samples. A negative correlation was frequently observed between the abundance of *Staphylococcus* and *Malassezia* OTUs, suggesting that they are antagonistic to each other in the dermal environment. AK may disorganize the epidermal lipids, leading to a decreased relative abundance of sebum-dependent *Propionibacterium* and *Malassezia* species, thus disrupting the microbiome homeostasis and exacerbating this condition.

## Conclusions

As one of the most common human cancer types, cSCC accounts for 20% of all skin tumor cases. The current main treatment method is excision of the tumor tissue and complete histological evaluation of the surgical margins. However, additional research is needed in SOT patients, as well as in patients with cSCC exhibiting primary or acquired resistance to immunotherapy.

In recent years, the interactions between skin microbiota dysbiosis and immunity have been increasingly investigated. However, the effects of these interactions on immune microecology and the development and progression of cutaneous malignancies remain largely unexplored. Therefore, an in-depth understanding of the specific mechanisms of these three-way interactions is a new direction for future academic research, as well as for clinical treatment.

The beneficial effects of probiotics have been intensively studied in recent years. Although little is known about the underlying mechanisms, probiotics have a clear impact on immune microecology and play a corresponding role in skin cancers, as mentioned above, they can suppress disease-causing microorganisms by participating in competition, or secreting specific metabolites [78–81], and can also affect the immune system and reduce inflammatory responses [78]. Therefore, further in vitro and in vivo studies are required to fully understand the properties of probiotics in the skin cancer field.

## Data Availability

No datasets were generated or analysed during the current study.

## Declarations

### Third party material

No, all of the material is owned by the authors and/or no permissions are required.
